# A case of synchronous serous ovarian cancer and uterine serous endometrial intraepithelial carcinoma

**DOI:** 10.1186/s13048-021-00835-8

**Published:** 2021-06-29

**Authors:** Maho Shimizu, Keitaro Yamanaka, Maho Azumi, Masako Tomimoto, Keiichi Washio, Ryosuke Takahashi, Satoshi Nagamata, Yuka Murata, Yui Yamasaki, Yoshito Terai

**Affiliations:** Department of Obstetrics and Gynecology, Kobe University Graduate School of Medicine, 7-5-2 Kusunoki-cho, Chuo-ku, Kobe, Hyogo 650-0017 Japan

**Keywords:** Serous ovarian cancer, Serous endometrial intraepithelial carcinoma, SEIC

## Abstract

**Background:**

Serous endometrial intraepithelial carcinoma (SEIC) is now considered to represent an early stage of uterine serous carcinoma (USC). It is an intraepithelial lesion but has been reported to cause extrauterine metastases. We report a case of SEIC with serous ovarian carcinoma and lymph node metastasis.

**Case presentation:**

A 57-year-old post-menopausal woman (gravida 3, para 2, SA1) was referred to our hospital with lower abdominal pain. An ultrasound and MRI showed that the ovary had swollen to 8 cm in size and had a solid lesion. The uterus was normal. The patient underwent exploratory laparoscopy on the suspicion of torsion of the ovarian tumor. Intraoperative findings showed a right ovarian tumor, but no ovarian tumor torsion was observed. A small amount of bloody ascites was found in the Douglas fossa, and bleeding was observed from the tumor itself. A right salpingo-oophorectomy was then performed. Histopathological results revealed a high-grade serous carcinoma. Forty days after the first surgery, we performed a staging laparotomy: a total abdominal hysterectomy, left salpingo-oophorectomy, systematic pelvic and paraaortic lymphadenectomy, and a partial omentectomy. A complete cytoreduction was achieved. In the pathological examination, the invasion of the serous carcinoma was observed in the left ovarian ligament, and lymph node metastasis was found in the paraaortic lymph nodes. Atypical columnar cells formed irregular papillary lesions which had proliferated in the endometrium, and this was diagnosed as SEIC. The final diagnosis was serous ovarian cancer, FIGO stage IIIA1(ii), pT2bN1M0, with SEIC.

**Conclusion:**

We report a case of SEIC with synchronous serous carcinoma of the adnexa uteri. Both were serous carcinomas and, thus, it was difficult to identify the primary lesion. The distinction between metastatic cancer and two independent primary tumors is important for an accurate diagnosis and tumor staging. Histological diagnostic criteria remain controversial, and further development of a method for differentiating between both diseases is required.

## Introduction

Serous endometrial intraepithelial carcinoma (SEIC) is now considered to represent an early stage of uterine serous carcinoma (USC) [1]. USC is an aggressive histologic subtype of endometrial cancer that has distinct clinical and pathologic characteristics, and it accounts for a disproportionate number of recurrences and deaths. Endometrioid adenocarcinoma usually occurs in perimenopausal or early postmenopausal women, is usually estrogen-dependent, develops in the background of endometrial hyperplasia, and has a generally favorable prognosis. In contrast, USC generally occurs in older women, is estrogen-independent, develops in the background of atrophic endometriosis, and often has a poor prognosis [2]. Despite minimal uterine involvement, SEIC has been associated with extrauterine metastasis. In a stage-matched study, there was no significant difference in the prognosis between SEIC and serous adenocarcinoma [3]. SEIC, whether limited to the endometrium or widely metastatic, should be treated comparably with USC. We report a case of SEIC with serous ovarian carcinoma and lymph node metastasis.

## Case presentation

**Fig. 1:**
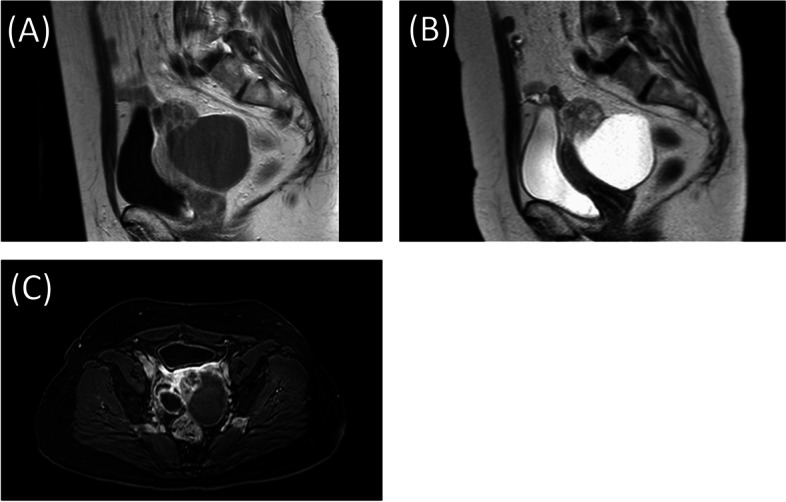
**A** T1-weighted MRI (**B**) T2-weighted MRI (**C**) Enhanced MRI. MRI with a 8.7 × 7.2 cm cystic and solid mass behind the uterus. A cystic lesion with a solid tumor was in the right adnexa. The solid lesion was well enhanced

**Fig. 2:**
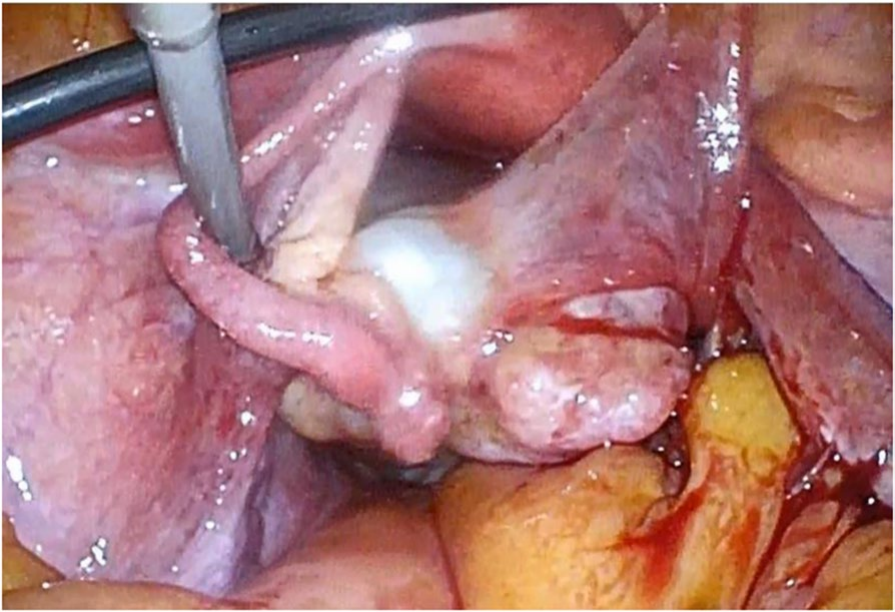
This intraoperative picture shows the ovarian mass with an irregular solid lesion infiltrating the right tubal fimbria and mesentery

**Fig. 3:**
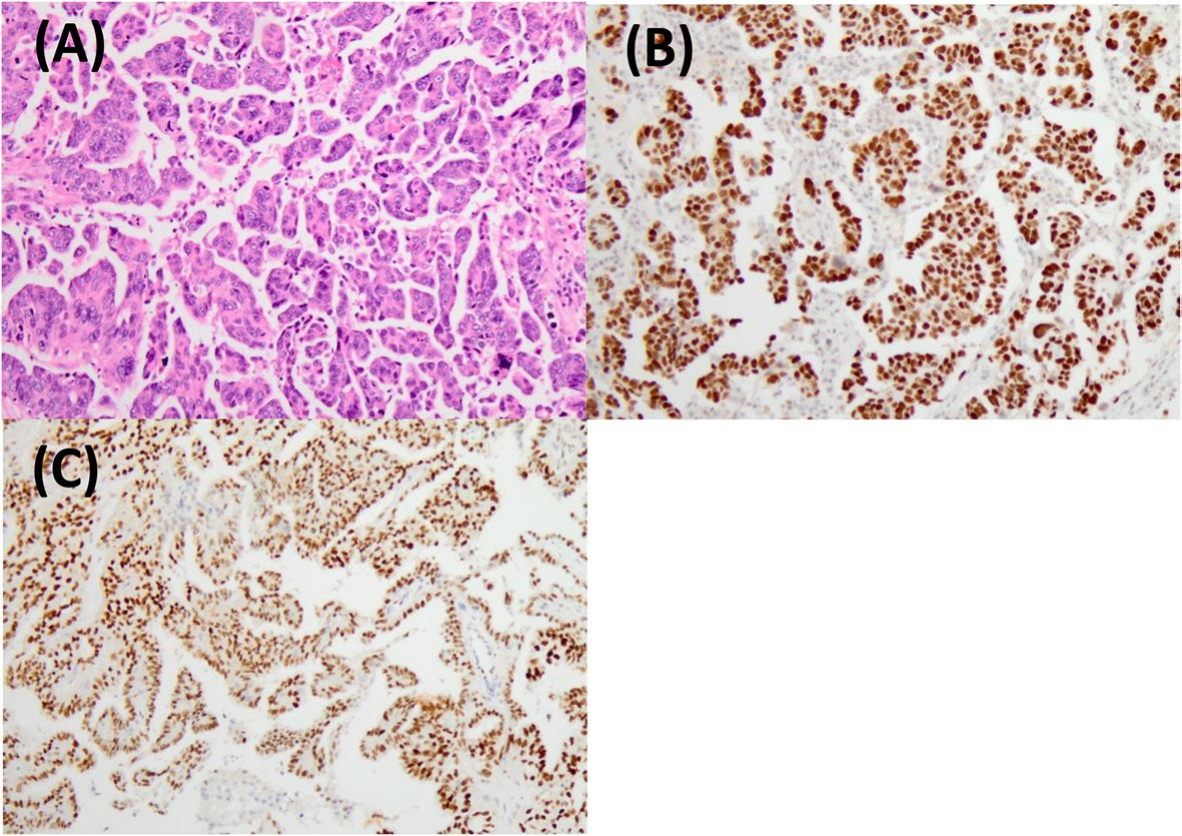
**A** Hematoxylin and eosin staining showed that, in the solid lesion of the ovary, atypical columnar cells had proliferated irregularly in a papillary manner. **B** p53-immunostaining was strongly positive, and **C** WT-1 immunostaining was diffusely positive

A 57-year-old post-menopausal woman (gravida 3, para 2, SA1) was referred to our hospital with intermittent lower abdominal pain. She had undergone an appendectomy in her twenties. Upon physical examination, her abdomen was soft, she had tenderness at the right lower-abdominal quadrant, and no mass was felt at the time. The patient’s routine blood analysis and renal function test were within normal limits: hemoglobin, 13.3 g/dL; total leukocytes count, 7.4 × 103/mm3, with 75% neutrophils, 19% lymphocytes, 4% monocytes and 1.2% eosinophils in the differential count; and platelet count, 210,000/mm3. Her serum level of urea nitrogen was 9.7 mg/dL, and creatinine was 0.75 mg/dL. The total bilirubin (1.8 mg/dL), alanine transaminase (56U/L), and aspartate aminotransferase (49U/L) were all mildly elevated, and her C-reactive protein was elevated to 10.8 mg/dl. Moreover, the patient’s serum level of cancer antigen 125 (CA-125) was elevated at 329/mL, and her serum levels of cancer antigen 19–9 (CA19-9) and Carcinoembryonic antigen (CEA) were 15 IU/L and 2.1 ng/mL, respectively, and both were within normal values. Upon ultrasonography, the right adnexa showed a cystic mass of 8.2 cm × 6 cm with a solid lesion. The uterus was normal in size and the endometrium was thin. Magnetic resonance imaging (MRI) revealed a 8.7 × 7.2 cm cystic and solid mass behind the uterus. The cystic part showed a homogeneous low intensity on T1-weighted MRI and heterogeneous high intensities on T2-weighted MRI; it was also well enhanced on contrast-enhanced MRI. The uterus was normal in size and the adnexa on the opposite side were unremarkable (Fig. 1). Pain in the lower-right abdomen persisted, and ovarian tumor torsion could not be ruled out. The patient then underwent an exploratory laparoscopy. A cystic lesion with a solid tumor in the right ovary (8 cm in the longest diameter) was found and had infiltrated the right tubal fimbria and mesentery in the pelvis (Fig. 2). No ovarian tumor torsion was observed, and no disseminated lesion was found in the peritoneal cavity. A small amount of bloody ascites was found in the Douglas fossa, and bleeding was observed from the tumor itself. A right salpingo-oophorectomy was then performed. The part of the mesentery that had adhered to the tumor was also resected. Histopathological results revealed a high-grade serous carcinoma in the ovary and fallopian tube, as well as in the mesentery, and immunostaining revealed strongly positive staining for p53 and diffusely positive staining for WT-1 in the serous carcinoma components (Fig. 3). A region in which atypical cells had proliferated was found in the ovary, and ovarian cancer was thus diagnosed.

**Fig. 4:**
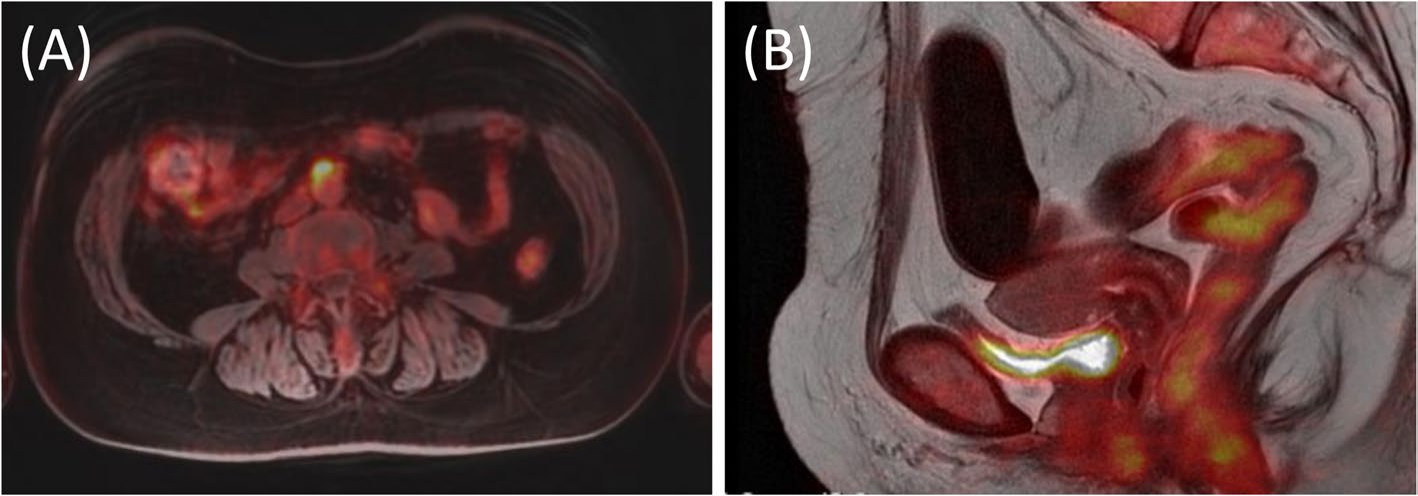
**A** On a PET-MRI, enlarged paraaortic lymph nodes with FDG accumulation were detected. **B** No obvious mass lesion was observed in the uterus

**Fig. 5:**
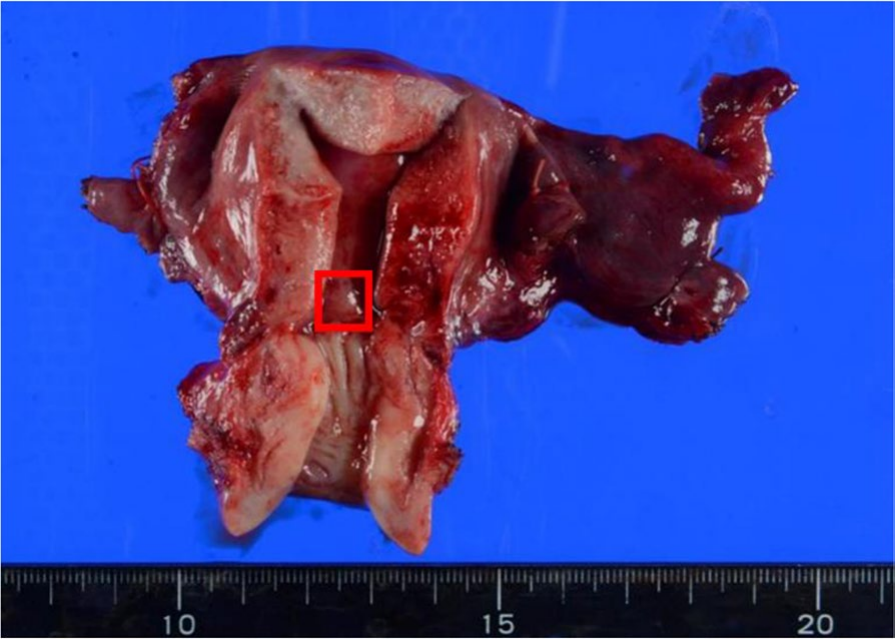
Macroscopically, a small elevated lesion was found in the uterine endometrium

**Fig. 6:**
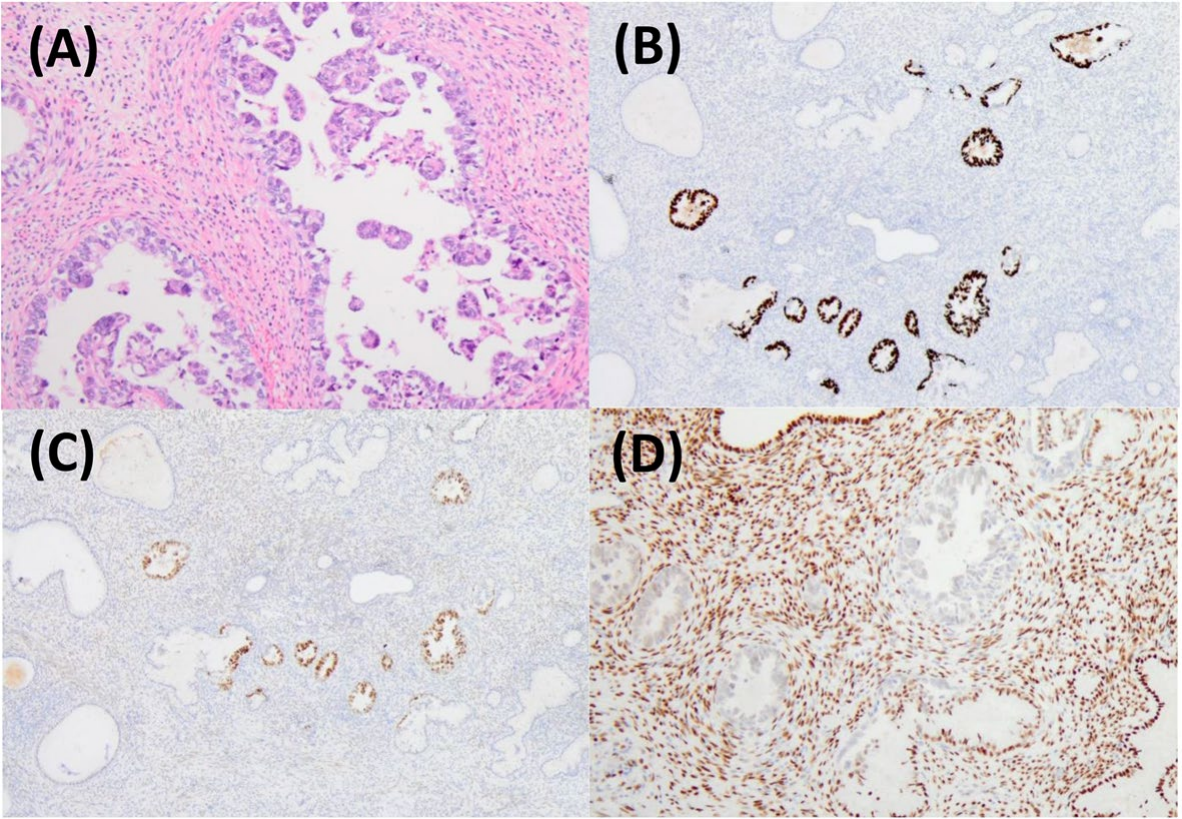
**A** Hematoxylin–eosin (HE) staining of the SEIC in the atrophic endometrium. Atypical columnar cells formed irregular papillary lesions and proliferated. **B** p53-immunostaining and **C** WT-1 immunostaining were positive. **D** ER immunostaining was negative

Upon a PET-MRI at a later date, enlarged paraaortic lymph nodes with FDG accumulation were detected (Fig. 4), and no obvious mass lesions were observed in the uterus. Forty days after the first surgery, we performed a staging laparotomy: a total abdominal hysterectomy, left salpingo-oophorectomy, systematic pelvic and paraaortic lymphadenectomy, and a partial omentectomy. A complete cytoreduction was achieved. Macroscopically, a small elevated lesion was found in the uterine endometrium (Fig. 5). Pathological examination revealed that atypical columnar cells formed irregular papillary lesions and had proliferated in the elevated lesion. There was no stromal invasion or myometrial invasion by the tumor cells. Immunostaining for p53 and WT-1 were positive, estrogen receptor (ER) was negative in the endometrial cells, and the pathological diagnosis of SEIC was then made (Fig. 6). Invasion of the serous carcinoma was observed in the left ovarian ligament. Although there were no lymph node metastases found in the pelvic lymph nodes (0/33), metastases were found in the paraaortic lymph nodes (10/29). The final diagnosis was serous ovarian cancer, FIGO stage IIIA1(ii), pT2bN1M0, with SEIC. The postoperative course was unremarkable and with no major complications. The patient received six courses of carboplatin, paclitaxel and bevacizumab as a postoperative adjuvant chemotherapy and, afterward, bevacizumab as a maintenance therapy was continued. At the time of this writing, nine months have passed since the operation, and there has been no evidence of recurrence.

## Discussion

SEIC is morphologically identified by the replacement of endometrial epithelium or glands with malignant cells that resemble endometrial serous carcinoma with high grade nuclei [1].

SEIC is characterized by the transition from atrophic endometrium to carcinoma in situ, and it is an immediate precursor of invasive uterine serous carcinoma (USC) [4]. USC is an aggressive histologic subtype of endometrial cancer and tends to have deep infiltration into the myometrium and early extrauterine spread. It also has a poor overall prognosis. USC is frequently found with a background of SEIC [5]. Immunohistochemical stains, such as p53 and estrogen receptor status, have been described in the literature and can identify these tumors.

Even though the tumor is localized to the endometrium, metastases outside the uterus are often observed. It is reported that 36–60% of patients with SEIC who underwent surgical staging had extrauterine metastatic disease [6, 7]. The absence of myometrial invasion is not predictive of the absence of lymph node or extrauterine metastases. Surgical staging for women with SEIC is important because those lesions frequently are associated with metastatic disease, despite any minimal uterine disease.

The finding of SEIC without coincident USC is uncommon [8, 9]. In this article, we report a case of SEIC with synchronous serous carcinoma of the adnexa uteri. Both were serous carcinomas and, thus, it was difficult to identify the primary lesion. No obvious findings in the uterus could be detected in the preoperative examination.

In synchronous female genital tract neoplasms, the combination of endometrial cancer and ovarian cancer accounts for 80% [10]. Since both neoplasms are often disseminated at diagnosis, and USC often has ovarian metastases, it may be difficult to identify the primary site. Pathological criteria play an important role in the differential diagnosis, but they can also cause a diagnostic dilemma. The immunophenotypic differences between ovarian serous carcinomas (OSCs) and USCs are subtle. Recently, it has been reported that Wilms tumor gene 1 (WT-1) may assist in the distinction of OSCs and USCs, since WT-1 staining patterns differ between these two carcinomas. It is reported that 80–97% of OSCs were reactive for WT-1, while 20–49% of USCs were found to be WT-1 positive [11–14]. ER reactivity is also reported to be useful for distinguishing between the two. ER reactivity is demonstrated in 64% of ovarian serous carcinomas, whereas only 11% of uterine serous carcinomas are ER positive [14].

In this case, WT-1 was positive and ER was negative, so it was difficult to determine which should have been the primary focus. Histological diagnostic criteria remain controversial, and the distinction between a metastatic carcinoma and two independent primary tumors is difficult. Since it is related to treatment and prognosis, future development of new diagnostic criteria is expected.

## Conclusion

We have described a case of SEIC with synchronous ovarian serous carcinoma. In this case, we could not determine whether SEIC antedated or merely coexisted with the extrauterine disease. The distinction between metastatic cancer and two independent primary tumors is important for accurate diagnosis and tumor staging. Histological diagnostic criteria remain controversial, and further development of a method for differentiating between both diseases is required.

This case revealed that the presence of SEIC should prompt an evaluation for an invasive USC and extrauterine disease. Also, when considering primary ovarian cancer, it is advisable to carefully examine the endometrium, in addition to the ovaries and fallopian tubes.

## Data Availability

The data used or analyzed are all included in this published article.
